# Medium-Sized Ring Expansion Strategies: Enhancing Small-Molecule Library Development

**DOI:** 10.3390/molecules29071562

**Published:** 2024-03-31

**Authors:** Hwiyeong Lee, Jonghoon Kim, Minseob Koh

**Affiliations:** 1 Department of Chemistry and Chemistry Institute for Functional Materials, Pusan National University, Busan 46241, Republic of Korea; gnldud1231@pusan.ac.kr; 2 Department of Chemistry and Integrative Institute of Basic Science, Soongsil University, Seoul 06978, Republic of Korea; jhkim19@ssu.ac.kr

**Keywords:** diversity-oriented synthesis, medium-sized ring synthesis, natural product mimics, ring-expansion reactions, bioactive compound discovery

## Abstract

The construction of a small molecule library that includes compounds with medium-sized rings is increasingly essential in drug discovery. These compounds are essential for identifying novel therapeutic agents capable of targeting “undruggable” targets through high-throughput and high-content screening, given their structural complexity and diversity. However, synthesizing medium-sized rings presents notable challenges, particularly with direct cyclization methods, due to issues such as transannular strain and reduced degrees of freedom. This review presents an overview of current strategies in synthesizing medium-sized rings, emphasizing innovative approaches like ring-expansion reactions. It highlights the challenges of synthesis and the potential of these compounds to diversify the chemical space for drug discovery, underscoring the importance of medium-sized rings in developing new bioactive compounds.

## 1. Introduction

In the field of drug discovery, the unique structural diversity and complexity of natural-product small-molecule libraries are exceptional, making them vital for discovering new bioactive compounds and developing drugs [1]. These natural products often exhibit distinctive biological activities not found in synthetic molecules, highlighting the potential of natural-product-based compounds for future therapeutic breakthroughs [2]. Unfortunately, the synthesis of natural products is hindered by their complex structures, leading to difficulties in constructing diverse natural products or natural-product-like small-molecule libraries [3]. This complexity prevents us from fully exploiting the therapeutic potential of natural products, emphasizing the need for novel approaches to synthesize and use these compounds more effectively in drug discovery.

To address the challenges in natural-product synthesis, diversity-oriented synthesis (DOS) has emerged as a key strategy for the efficient construction of libraries via the creation of numerous small molecules through diverse synthetic pathways [4,5,6]. This approach is particularly effective for exploring the extensive chemical space of natural products and natural-product-like molecules, leading to the discovery of unique compounds with potential biological activities [7,8]. Natural-product-like DOS libraries comprise various synthetic compounds comparable to collections of natural products in terms of complexity and diversity, which have been demonstrated through the identification of various bioactive compounds in undruggable targets and unbiased phenotypic screening [9,10,11].

**Figure 1:**
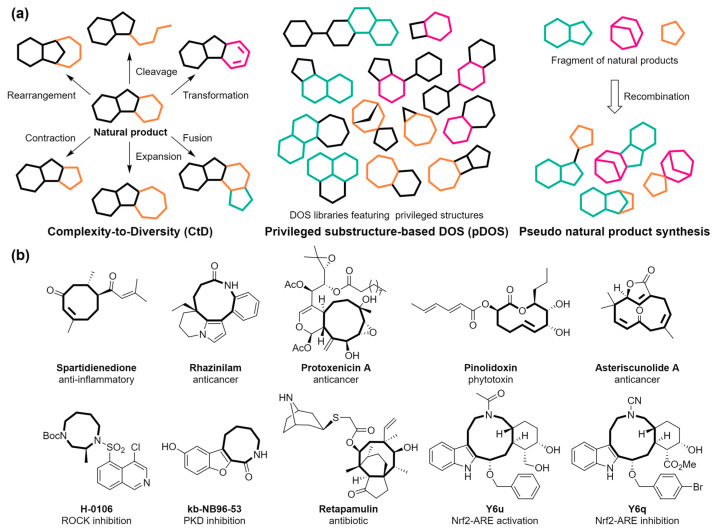
(**a**) Schematic representation of complexity-to-diversity (Ctd) strategy, privileged substructure-based DOS (pDOS), and pseudo-natural products synthesis approach. In Ctd, the rings undergo transformation and are distinguished by various colors. The colored rings in pDOS and pseudo-natural product synthesis refer to privileged structures and fragments derived from natural products, respectively. (**b**) Representative examples of natural and bioactive compounds with medium-sized rings [48,49,50,51,52,53,54,55].

Despite the success of DOS strategies in constructing natural-product-like compound libraries, there remains a demand for complementary synthetic approaches for various natural products and related compounds with varying structural diversities and complexities. This has inspired significant research into novel approaches, such as complexity-to-diversity (Ctd), biomimetic synthetic strategies, biology-oriented synthesis (BIOS), pseudo-natural products synthesis, and privileged substructure-based DOS (pDOS), aiming to enhance the diversity and complexity of small-molecule libraries with high biological relevancy (Figure 1a).

The Ctd approach begins with natural products, using their complex structures to systematically generate structurally diverse molecules with high biological relevancy [12,13,14,15,16]. This strategy exploits the rich complexity of natural compounds to synthesize various molecular structures, which allows for populating unexplored natural-product-based chemical space. In contrast, biomimetic synthetic strategies mimic the efficient pathways found in biological systems, aiming to apply these efficient pathways to produce new molecules with potential biological or therapeutic benefits [17,18,19,20]. Both methods represent a shift toward more versatile methods of synthesis in drug discovery, aiming to explore and harness the unexplored chemical spaces of natural products, thereby successfully expanding the scope of molecules available for therapeutic development.

BIOS employs a synthetic strategy to construct compound libraries inspired by bioactive natural products [21,22,23,24]. Unlike traditional methods that aim for broad chemical diversity, BIOS specifically targets molecules with structures known to effectively interact with biological systems. This methodology relies on the hypothesis that certain molecular frameworks observed in nature have evolved to interact with specific biological targets, making them valuable candidates for drug discovery. Thus, BIOS rationalizes therapeutic discovery by targeting a narrower chemical space with proven biological significance, focusing on molecular structures already validated through biological activity.

Pseudo-natural products represent a novel class of compounds inspired by natural products but constructed through the chemical combination of biosynthetically unrelated natural product fragments [25,26,27]. This approach aims to synthesize compounds that possess the biological relevance and diversity of natural products while exploring new chemical spaces and potential biological activities not observed in nature. The recombining fragments from different natural products in novel arrangements allow for the generation of distinctive structures. This method holds the promise of uncovering previously unknown biologically active compounds.

pDOS is an advanced strategy for constructing libraries of polyheterocyclic compounds with high biological relevance [28,29,30]. This approach utilizes privileged substructures, i.e., molecular frameworks known to effectively interact with multiple types of biological targets, to efficiently explore new biologically relevant chemical spaces. By incorporating these privileged substructures into polyheterocyclic core skeletons, pDOS aims to generate many compounds that specifically interact with biopolymers, enhancing the discovery of bioactive molecules with significant specificity and potential for therapeutic applications. This approach demonstrates the value of pDOS libraries and highlights their role in discovering diverse small-molecule modulators across various undruggable biological targets [31,32,33,34].

Despite significant progress in constructing libraries of small molecules resembling natural products, the synthesis of medium-sized rings, such as those with 8 to 11 members, is notably challenging due to the unique strain and structural constraints they exhibit. One of the primary obstacles in synthesizing these structures is transannular strain, a consequence of the proximity of atoms or functional groups within a ring, which are not directly bonded to each other but are close enough to cause repulsive interactions [35,36,37,38,39,40,41]. This strain crucially influences the conformational stability and preferences of medium-sized rings, affecting their energy and, consequently, their reactivity and chemical behaviors [42]. Medium-sized rings, with structural diversity similar to that found in natural products, exhibit significant potential for application in drug discovery because of their unique structural characteristics (Figure 1b). However, they are notably lacking in current screening libraries, leading to their limited presence among top-selling pharmaceuticals. Thus, exploring new bioactive chemical spaces through medium-sized ring synthesis is essential for discovering novel bioactive compounds.

Ring-expansion reactions of polycyclic substrates are key to synthesizing medium-sized rings, enabling the efficient and practical creation of various molecular structures that cannot be achieved through direct cyclization due to enthalpic and entropic factors [43,44,45,46,47]. This approach allows chemists to efficiently synthesize medium-sized rings, which are essential for drug development and the construction of medium-sized ring-based small-molecule libraries, by efficiently transforming smaller rings or linear precursors. The process, designed to address thermodynamic barriers, generally employs reactive intermediates to yield thermodynamically stable products. This not only enhances the structural diversity available for pharmaceutical research but also addresses the synthetic challenges associated with medium-sized ring compounds. This review spotlights the recent and significant developments in ring-expansion synthetic methodologies, emphasizing the critical contribution of medium-sized rings for diversifying the chemical space. We classify these methods by their mechanisms and the specific polycyclic substrates they work on. Covering research from 2010 to the current day, we aim to highlight the transformative progress in the synthesis of medium-sized rings, showcasing their growing importance and potential in the last decade.

## 2. Ring-Expansion Reactions via the Cleavage of the C–C Bond for the Synthesis of Medium-Sized Rings

**Scheme 1:**
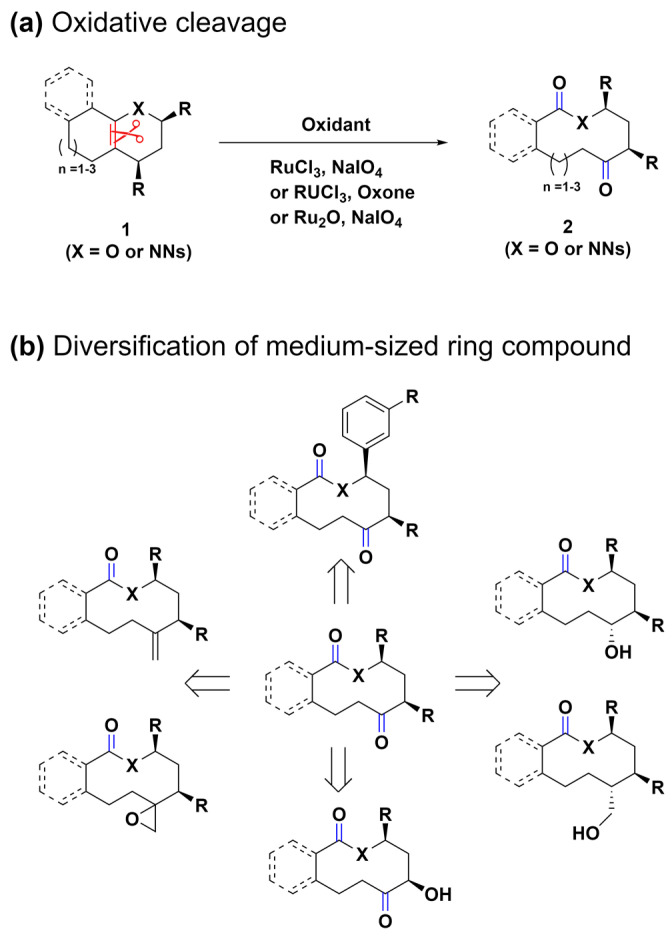
(**a**) Ring-expansion reactions for the synthesis of medium-sized lactones and lactams via the oxidative cleavage of the C=C double bond (highlighted in red). (**b**) Diversification of the medium-sized ring compound.

The exploration of methods to selectively cleave C–C single or C=C double bonds in polycyclic precursors offers a promising route for synthesizing medium-sized rings. Tan et al. developed a novel approach for the diverse synthesis of medium-ring lactones and lactams **2** via the oxidative cleavage of the C=C double bond (highlighted in red)-bridged polycyclic compounds **1** (Scheme 1a) [56]. This method efficiently addresses the limitations of traditional cyclization strategies, which are often restricted by ring size and substrate stereochemistry. Noteworthy for its mild conditions, this synthetic method facilitates the integration of various functionalities into macrocyclic structures. This, in turn, paves the way for further structural diversification and functionalization, significantly broadening the scope for constructing libraries of medium-sized ring compounds (Scheme 1b). Such libraries are invaluable for drug discovery and the construction of natural-product libraries, offering systematic access to a diverse array of medium-ring frameworks.

**Scheme 2:**
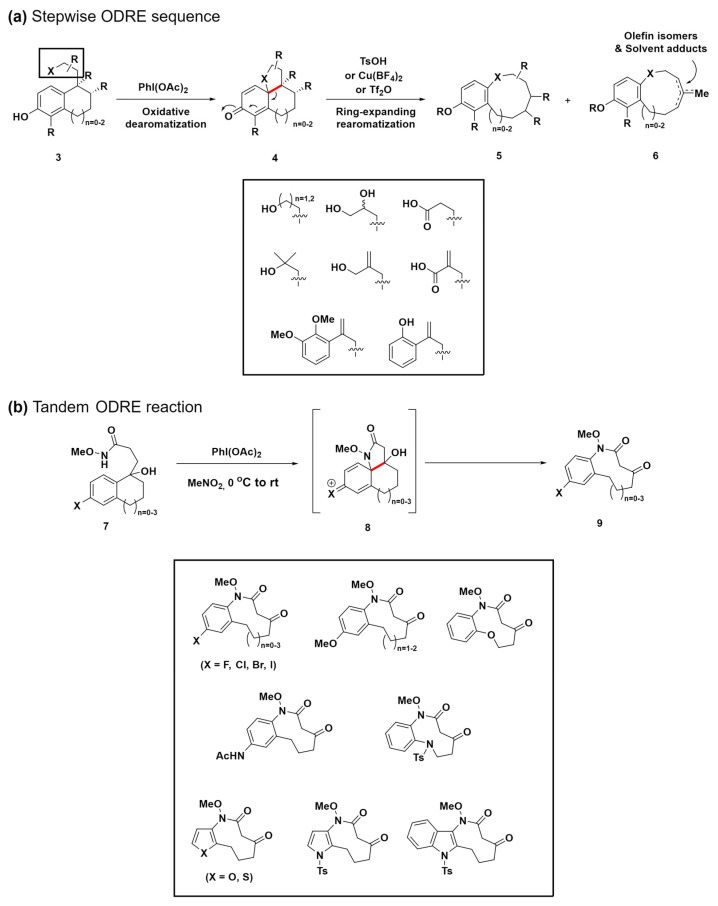
(**a**) Biomimetic ODRE stepwise sequence for benzannulated medium-ring synthesis. (**b**) Tandem ODRE reaction for diverse benzannulated medium-ring lactam synthesis. C–C bond cleavage was highlighted in red.

Ring-expansion reactions, which are pivotal in biosynthetic pathways for generating medium-ring natural products, present a novel method for the creation of complex structures [57,58]. Inspired by these natural processes, the same research group devised a biomimetic, diversity-oriented synthetic strategy for the construction of benzannulated medium rings (Scheme 2a) [59]. Through the oxidative aromatization of bicyclic phenol compounds **3**, polycyclic cyclohexadienone intermediates **4** were synthesized. These intermediates **4** undergo a strategic aromatization-driven ring expansion, effectively cleaving scissile bonds (highlighted in red) under conditions that are carefully optimized to suppress side reactions, such as dienone–phenol rearrangement. This oxidative dearomatization–ring expansion–rearomatization (ODRE) sequence proficiently generates various medium-sized ring scaffolds, employing a wide array of nucleophiles, including acids, aryls, phenols, primary alcohols, and tertiary alcohols.

Notably, in 2018, the same research group refined this approach through a tandem ODRE reaction that employs the umpolung strategy (Scheme 2b) [60]. Although the previous ODRE sequence yielded diverse ring linkages found in medium-ring natural products, such as aryl ethers, diaryl ethers, lactones, and biaryls, it was primarily applicable to phenolic substrates. Additionally, this previous approach occasionally resulted in the formation of olefin isomer mixtures and solvent adduct compounds **6** due to various termination reactions, limiting its application despite its broad applicability (Scheme 2a). To enhance versatility beyond phenolic substrates and address the various issues, the novel umpolung strategy employs an electron-rich aromatic ring to target an electrophilic side chain, resulting in the formation of a cationic tricyclic intermediate **8**, enabling direct ring expansion through a tandem process. This strategy also introduces tertiary alcohol into the substrate, which leads to a ketone product while avoiding alternate termination pathways, thus offering a versatile tool for further functionalization. This method expands the range of substrates, facilitating the synthesis of crucial components, such as haloaryl, aryl ether, acetanilide, aryl sulfonamide, and heteroaromatic medium-ring products. These products are key to numerous natural and synthetic pharmacophores, representing a notable advancement in the synthesis of medium-sized rings. This progress opens up new possibilities for drug discovery and the development of diverse chemical libraries.

**Scheme 3:**
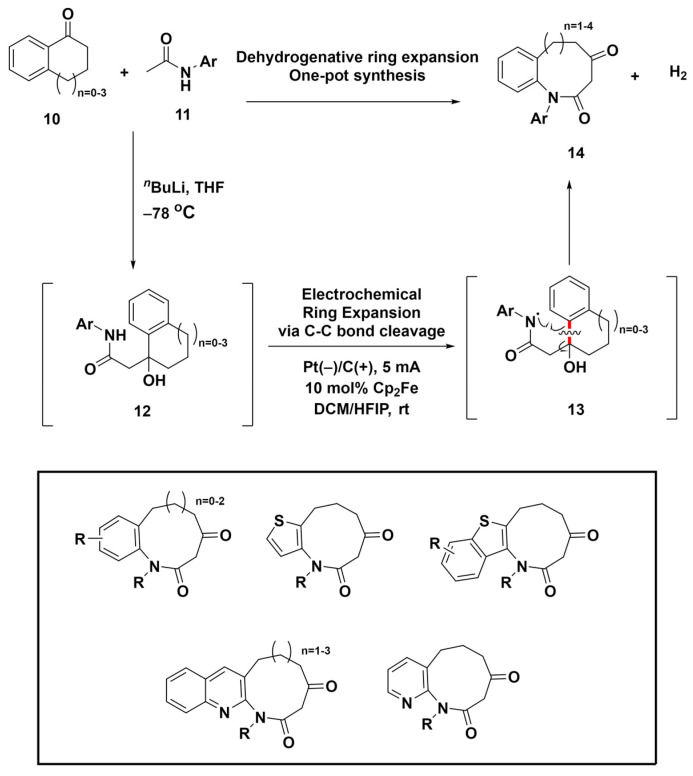
Electrochemical ring expansion for the synthesis of diverse privileged-structure-embedded medium-sized ring compounds via C–C bond cleavage (highlighted in red).

Electrochemical oxidation has emerged as a safe, eco-friendly alternative to conventional oxidants, offering precise reaction control [61,62,63]. This sustainable technique facilitates the creation of reactive intermediates from organic compounds via direct or indirect electrolysis, sparking interest in the electrochemical assembly of medium-sized rings. In 2020, Liu et al. introduced a versatile approach for effective dehydrogenative ring expansion, enabling the synthesis of medium-sized lactams through amidyl radical migration-induced C–C bond cleavage (Scheme 3) [64]. Using readily available benzocyclic ketones **10** and amides **11**, this strategy produces eight- to eleven-membered lactam compounds **14** with notable efficiency and yield, demonstrating significant tolerance to diverse substituents. The method relies on the migration of amidyl radicals, which induces the cleavage of the C–C bond (highlighted in red), followed by a single-electron oxidation step. This sequence provides a reliable route to medium-sized lactams, which are essential components of bioactive compounds and natural products. Additionally, various privileged structure-embedded compounds within medium-sized rings were successfully synthesized using this method, as shown in Scheme 3. This advancement not only overcomes the synthetic hurdles due to unfavorable enthalpic and entropic barriers but also provides new routes for the formation of various medium-ring compounds.

## 3. Ring-Expansion Reactions via the Cleavage of the N–N Bond for the Synthesis of Medium-Sized Rings

**Scheme 4:**
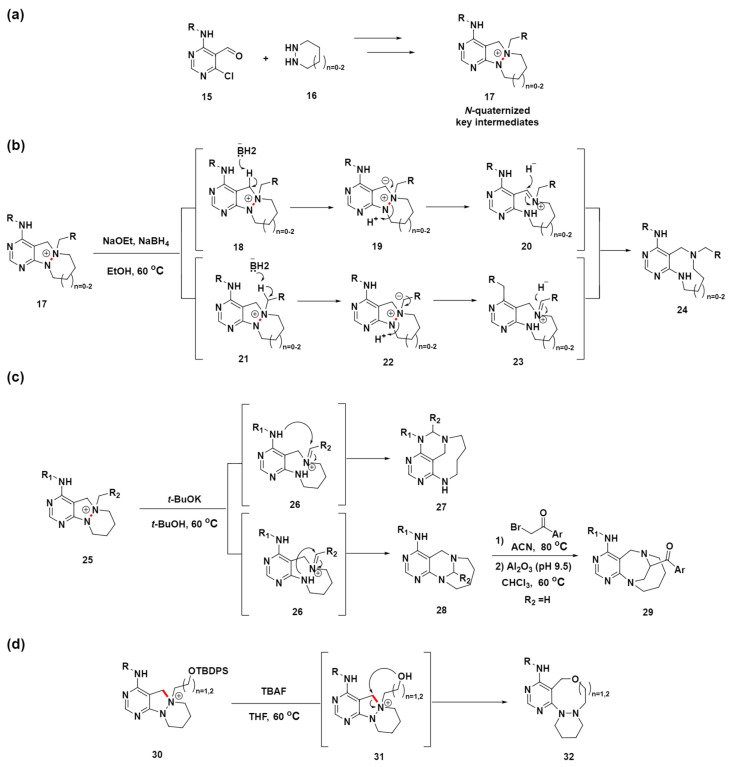
pDOS synthetic strategy for pyrimidine-embedded medium-sized ring compounds. (**a**) Design and synthesis of an *N*-quaternized key intermediate for the construction of a medium-sized small-molecule library containing a pyrimidine-privileged substructure. (**b**) Ring-expansion reactions for the synthesis of a medium-sized ring via the cleavage of the N–N bond. (**c**) Synthesis of a bridged medium-sized ring via the migration of an N–N–C bond to N–C–N. (**d**) Ring-expansion reactions for the synthesis of a medium-sized ring via internal nucleophile-driven C–N bond cleavage. N–N or C–N bond cleavage sites were highlighted in red.

In the previous section, we explored the selective cleavage of either a C–C single bond or a C=C double bond to generate medium-sized ring compounds from fused polycyclic precursors. This examination enabled the implementation of a series of DOS approaches and biomimetic synthetic strategies for creating diverse medium-sized molecular scaffolds. These approaches primarily utilized the cleavage of C–C single or double bonds, demonstrating a strategic pathway to efficiently achieve structural diversity in medium-sized rings. In comparison, the exploration of N–N bond cleavage to create medium-sized rings is less common. Moreover, the reductive cleavage reaction of N–N bonds for ring expansion is not conventionally mild, hindering the introduction of diverse functional groups for further diversification. In contrast, in 2019, Park et al. introduced an innovative approach utilizing N–N bond cleavage to effectively construct unique medium-sized azacycles with embedded pyrimidine, representing a notable breakthrough in this area [65]. This strategy involved the generation of key intermediates through the *N*-quaternization of azacyclic precursors **17**, achieved by the sequential cyclization and reduction of functionalized pyrimidine **15** with cyclic hydrazines **16**, followed by targeted *N*-quaternization (Scheme 4a). These inherently unstable intermediates undergo ring expansion through selective N–N bond cleavage (highlighted in red), facilitating the synthesis of medium-sized rings.

After optimizing the reaction conditions for the N–N bond cleavage, inspired by a previous study on N–N bond cleavage via Hofmann-type elimination [66], they discovered that using NaOEt and NaBH_4_ in ethanol effectively converts the *N*-quaternized intermediate **17** into medium-sized ring-fused pyrimidine compounds **24** (Scheme 4b). Notably, the reaction conditions were sufficiently mild to allow for the synthesis of a functionalized pyrimidine-fused medium-sized ring suitable for late-stage modification. Initially, they hypothesized that N–N bond cleavage occurred via Hofmann-type β-elimination; however, inconsistencies, such as the reaction’s failure with only NaOEt and the presence of more acidic benzylic protons, suggested a different mechanism. Further investigation, including a deuterium-labeling experiment, indicated that the cleavage might proceed through the formation of iminium species **20** or **23**, followed by hydride addition. This led us to propose a new mechanism for the N–N bond cleavage involving base-promoted iminium formation and subsequent neutralization by hydride.

To advance the synthesis of diverse pyrimidine-embedded medium-sized rings via N–N bond cleavage, an intramolecular reaction strategy was devised to convert an N–N–C bond into an N–C–N bond (Scheme 4c). Using a base, *t*-BuOK, and without a hydride source, the precursor compound **25** was transformed into two distinct medium-sized pyrimidine-fused products **27** and **28** through the formation of an iminium intermediate **26**, which then experienced an intramolecular nucleophilic attack from each anilinic nitrogen. In addition, the subsequent modification of compound **28** afforded novel homologated bridged medium-sized azacycles **29**. This was accomplished via selective *N*-quaternization, followed by migration under mild basic conditions.

In addition, a unique synthesis pathway for a different class of pyrimidine-embedded medium-sized rings was established, exploiting the cleavage of the C–N bond (highlighted in red) via internal nucleophilic attack (Scheme 4d). Specifically, compound **30**, designed with an allyloxy group as a potential nucleophile, was transformed into compound **32** with an expanded ring structure. This transformation was facilitated by the application of tetrabutylammonium fluoride to remove the silyl protective group, thereby allowing for an intramolecular nucleophilic attack by the alkoxide group. This pDOS strategy provides a new divergent synthetic pathway via an *N*-quaternizing strategy for efficient access to diverse pyrimidine-embedded medium-sized azacycles. This unique chemical library of medium-sized azacycles may serve as a valuable resource for the discovery of novel small-molecule modulators and potential therapeutic agents.

## 4. Ring-Expansion Reactions via the Cleavage of the C–N Bond for the Synthesis of Medium-Sized Rings

Methods for ring expansion through C–N bond cleavage are more widely known than other methods. Moreover, the discussion of C–N cleavage for the synthesis of diverse medium-sized rings is covered in several reviews [67,68,69]. Therefore, this review aims to concisely summarize recent divergent synthetic strategies, focusing on studies reported between 2020 and 2024.

**Scheme 5:**
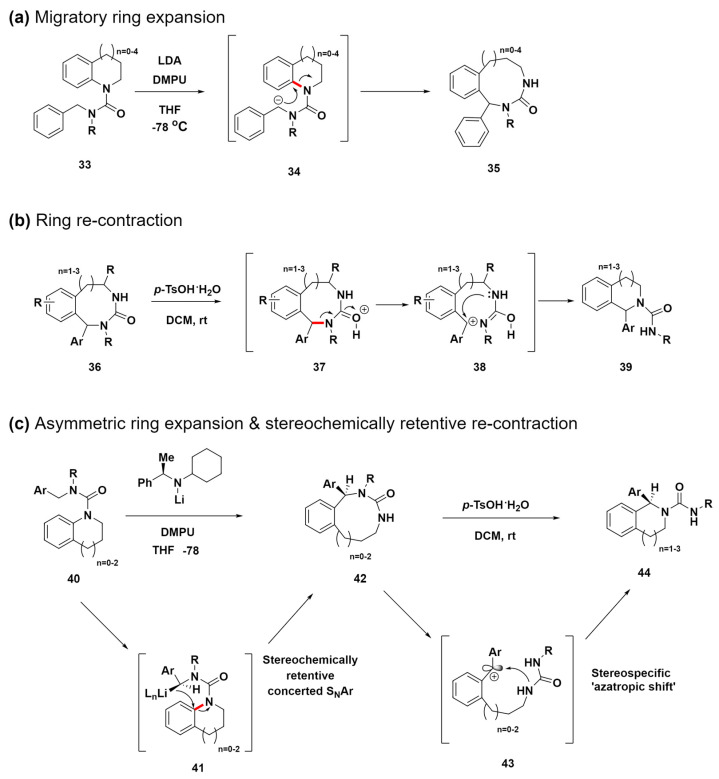
(**a**) Migratory ring-expansion reaction for the synthesis of medium-sized rings with a range of benzo-fused cyclic-urea structures. (**b**) Acid-promoted cyclic-urea ring contraction. (**c**) Asymmetric ring expansion for the synthesis of medium-sized rings and the stereochemically retentive recontraction of cyclic ureas. C–N bond cleavage sites were highlighted in red.

Clayden et al. developed a method for creating medium-sized nitrogen heterocycles (8- to 12-membered rings) via the migratory ring expansion of metallated ureas, a technique that efficiently yields benzodiazepines, benzodiazocines, and related benzo-fused nitrogen heterocycles (Scheme 5a) [70]. This strategy utilizes lithium diisopropylamide and *N,N′*-dimethylpropylideneurea for the selective deprotonation of compounds **33** to form anion intermediates **34**. This promotes a migratory ring expansion that transforms them into n + 3-membered product compounds **35** through a nucleophilic attack. This method not only efficiently synthesizes challenging medium-ring nitrogen heterocycles but also exhibits adaptability to various substituents, highlighting its potential for use in various synthetic processes.

Expanding upon the previous study, subsequent research introduces a novel approach using the acid-promoted ring contraction of cyclic ureas to synthesize complex scaffold compounds **39** (Scheme 5b) [71]. This process is initiated by the protonation of the electron-rich oxygen atom in the urea moiety of compound **36**, leading to a carbocation intermediate **38**. Subsequently, intermediate **38** undergoes an intramolecular S_N_1 substitution, where it is captured by the nitrogen atom in the urea. This key step facilitates ring contraction, ultimately yielding compound **39**. This novel approach demonstrates the high potential of using ring contractions in complex molecule synthesis.

In their 2024 study, they advanced the synthesis of benzo-fused nitrogen heterocycles via asymmetric ring expansion and the stereochemically retentive recontraction of cyclic ureas, overcoming the limitation of racemic products from previous methods (Scheme 5c) [72]. This innovative approach allows for the enantioselective synthesis of complex medium-sized ring structures using a chiral lithium amide base to initiate the asymmetric migratory ring expansion of ureas derived from indoline, tetrahydroquinoline, or tetrahydrobenzazepine. This study suggests that the enantioselective formation of benzyl lithium intermediates **41** upon treatment with a chiral lithium amide base occurs via a stereospecific intramolecular S_N_Ar reaction. This reaction facilitates enantioselective migratory ring expansion, finally yielding compounds **42**. This step is pivotal for the method to produce enantioenriched compounds effectively. Furthermore, the process involves a unique stereospecific ‘azatropic shift’ during the acid treatment of compounds **42**. This shift leads to a two-atom ring contraction to produce enantioenriched heterocyclic compounds **44** with remarkably retained stereochemistry. This technique advances the synthesis of enantioenriched medium-sized ring compounds, offering new avenues for drug discovery and the creation of libraries of medium-sized, natural product-like molecules.

**Scheme 6:**
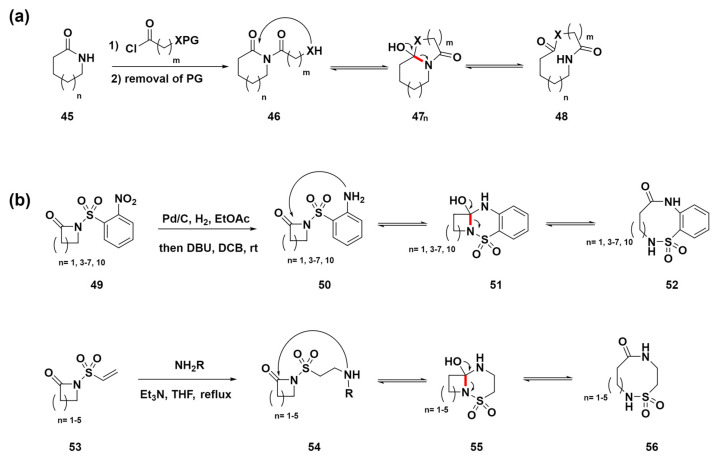
(**a**) Reaction sequence involving acylation, removal of protecting groups, and ring expansion to synthesize medium-sized lactams and lactones. (**b**) Strategies for expanding rings in the synthesis of medium-sized and macrocyclic sulfonamides. C–N bond cleavage sites were highlighted in red.

Unsworth et al. developed a novel method for synthesizing structurally diverse medium-sized to macrocyclic compounds through a ring-expansion reaction sequence (Scheme 6a) [73,74,75,76,77,78]. This advanced strategy incorporates steps such as acylation, protective group removal, and ring expansion to generate various lactams and lactone compounds **48**. This methodology has led to the creation of a variety of medium-sized ring compounds, demonstrating its immense potential in drug discovery for highlighting a flexible route to structures of medicinal relevance. Additionally, in 2023, they presented two novel ring-expansion methods for synthesizing medium-sized and macrocyclic sulfonamide compounds **52** and **58** (Scheme 6b) [79]. The key steps involve nitro reduction or amine conjugate addition to initiate ring expansion, producing diversely functionalized cyclic sulfonamides with good-to-excellent yields across various ring sizes. This method employs two novel strategies for the synthesis of medium-sized and macrocyclic sulfonamides without the need for protecting groups, highlighting its significantly improved synthetic accessibility. The initial strategy involved the nitro reduction of 2-nitro sulfonamide precursors **49**, which are easily derived from 2-nosyl chloride, to generate benzannulated products **52** through cyclization and ring expansion. An alternative pathway for non-benzannulated products employs vinyl sulfonamide precursors **53**, using conjugate addition with primary amines for cyclization and ring expansion to provide compounds **56**. These methods promise to expedite exploration in medicinal chemistry and other applied fields by offering straightforward routes to complex structures of relevance.

**Scheme 7:**
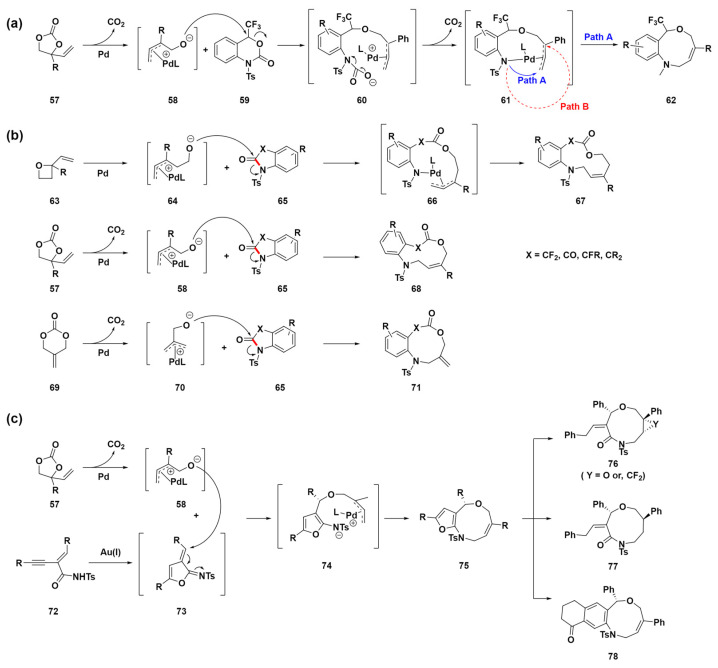
(**a**) Pd-catalyzed ring-expansion reaction of trifluoromethyl benzo[*d*][1,3]oxazinones **59** with vinyl ethylene **57** for the synthesis of nine-membered heterocyclic compounds **62**. (**b**) Synthesis of medium-sized heterocyclic lactones through sequential C–N bond cleavage (highlighted in red) and ring expansion. (**c**) Sequential catalysis system using gold and palladium to synthesize furan-fused nine-membered heterocycles.

In 2018, Shibata et al. developed a palladium-catalyzed double decarboxylative formal ring expansion to access nine-membered heterocyclic compounds **62** with a trifluoromethyl carbinol moiety [80]. Starting from six-membered trifluoromethyl benzo[*d*][1,3]oxazinones **59** and reacting with vinylethylene carbonates **57**, they achieved direct conversion to nine-membered trifluoromethyl benzo[*c*][1,5]oxazonines **62** (Scheme 7a). This method facilitates the direct synthesis of trifluoromethylated benzo[*c*][1,5]oxazonines by incorporating various functional groups within the aromatic unit, such as electron-donating, electron-withdrawing, and halogen groups. In addition, the method enables further functionalization of the alkene moiety in these products through processes such as epoxidation and reduction, achieving high diastereoselectivities. This enhancement of the trifluoromethylated nine-membered framework’s versatility underscores its potential as a promising scaffold in drug discovery research. The proposed mechanism involves the oxidative addition of Pd(0) to vinylethylene carbonates **57**, followed by decarboxylation, forming a π-allyl-Pd(II) complex intermediate **58**. This complex’s alkoxide oxygen, due to its high nucleophilicity, attacks the electrophilic carbon of trifluoromethylated benzo[*d*][1,3]oxazinones **59**, leading to ring opening and the formation of a highly reactive species **60** that undergoes decarboxylation to a Pd complex **61**. Two cyclization pathways could lead to different products, but only the [5 + 4] cycloaddition product **62** was observed, possibly due to steric hindrance preventing [4 + 3] cycloaddition.

In 2020, they also developed a novel method for synthesizing various medium-sized, fluorinated and nonfluorinated, heterocyclic lactones through a detailed process involving sequential C–N bond (highlighted in red) cleavage and ring expansion catalyzed by palladium (Scheme 7b) [81]. This method notably generated gem-difluoromethylene *N*-heterocyclic lactones ranging from 9- to 11-membered rings, employing difluoro-oxindoles **65** along with various zwitterion precursors **58**, **64**, or **70**. The compatibility of the gem-difluoromethylene group with prevalent pharmaceutical motifs enhances the significance of this synthetic method in drug development, particularly for the creation of medium-sized rings, which is valuable. Typically, heterocyclic compounds featuring gem-difluoromethylene are confined to four- to six-membered rings, which makes the synthesis of medium-sized variants a substantial challenge. To address this challenge, the study introduces a groundbreaking metal-catalyzed annulation strategy that employs a zwitterionic intermediate for C–N bond cleavage, opening up novel pathways for the assembly of complex molecules. This method notably uses vinyl oxetanes **63**, vinyl ethylene carbonates **57**, or 2-methylidenetrimethylene carbonates **69**, which are transformed into π-allyl-Pd(II) complex intermediates **64**, **58,** or **70**, respectively. For intermediates **64**, a nucleophilic oxygen anion targets the carbonyl group in oxindoles **65**, affording palladacycle intermediates **66**. This is followed by a reductive elimination step, which produces the desired annulation medium-sized ring products **67**. The same mechanism is used for the other intermediates **58** or **70**, facilitating the synthesis of medium-sized ring products **68** or **71**, respectively. This approach not only demonstrates versatility in integrating diverse functional groups and adjusting ring sizes but also exemplifies the significant advancements in the development of novel pharmacophores. Accordingly, this approach holds substantial application potential in DOS for the construction of a library of medium-sized ring molecules.

Although various π-allyl-Pd(II) complexes have offered researchers a path to synthesize a broader range of medium-sized ring compounds, previous reactions predominantly yielded benzofuran- or benzo-fused medium-sized heterocycles. To overcome this limitation, sequential or cascade catalysis has been implemented, enabling the efficient one-pot synthesis of complex structures through multistep processes. In 2021, Yang et al. introduced a method for the stereoselective synthesis of polyfunctionalized nine-membered heterocycles using sequential gold and palladium catalysis, as shown in Scheme 7c [82]. This method employs gold-catalyzed enyne cyclization, which is notably effective for the in situ generation of dipolarophiles. Further, this method facilitates dipolar cycloaddition, yielding furan-fused medium-sized heterocyclic compounds **75**. These compounds **75** can subsequently be functionalized further, allowing for the creation of a broad spectrum of both fused and non-fused medium-sized heterocyclic compounds **76**, **77**, and **78**. This represents a novel pathway for synthesizing medium-sized rings. The proposed mechanism starts with Pd reacting with vinyl ethylene carbonates **57** to form π-allyl-Pd(II) complex intermediates **58**. Next, the alkoxide moiety of the intermediates **58** undergoes an oxy-Michael addition to azadiene **73**. This taxadiene **73** is formed through the gold-catalyzed cyclization of enynamide **72**, resulting in the formation of the zwitterion intermediate **74**. Subsequently, a reductive elimination process occurs, leading to targeted annulation and yielding medium-sized ring products **75**. This sequence demonstrates a sophisticated interplay of catalytic cycles that facilitates the synthesis of complex structures. Despite the absence of C–N bond cleavage in this particular mechanism, the method highlights the potential of sequential or cascade catalysis to overcome the synthetic limitations commonly encountered in medium-sized ring formation. This example was chosen to illustrate the expanding toolkit available to researchers for the construction of complex medium-sized ring structures, emphasizing that the strategic application of π-allyl-Pd(II) complexes is crucial for advancing chemical diversity.

## 5. Ring-Expansion Strategies for Synthesizing Complex Natural-Product-like Molecules with Medium-Sized Rings

**Scheme 8:**
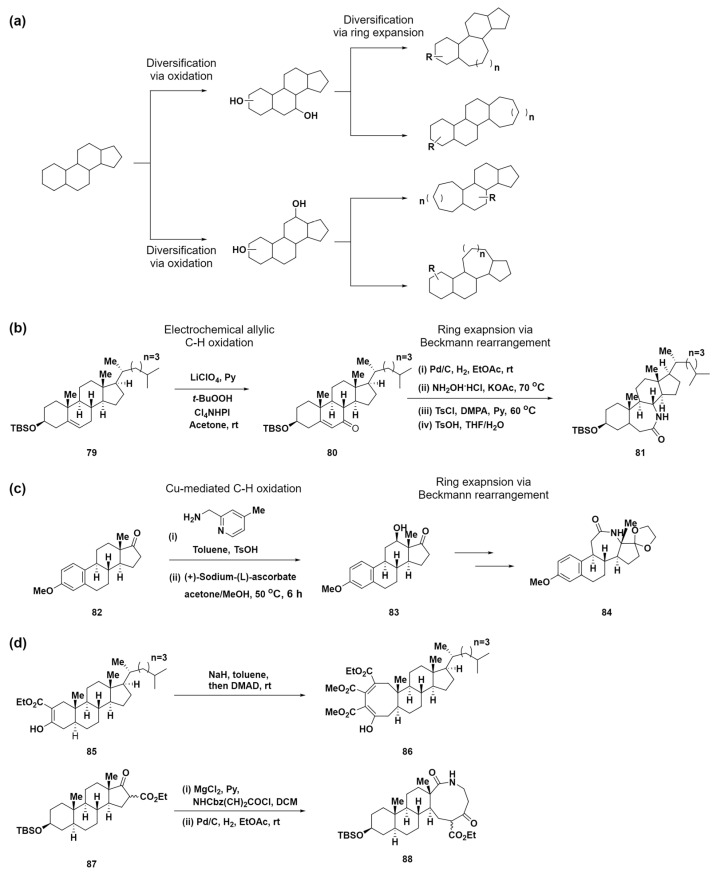
(**a**) Diversification strategy through site-selective C–H oxidation and subsequent ring expansion reaction. (**b**) Electrochemical approach for allylic C–H oxidation combined with Beckmann rearrangement to synthesize lactams with ring expansion. (**c**) Site-selective Cu-mediated C–H oxidation combined with a Beckmann rearrangement. (**d**) Complex structural transformations utilizing C–O bonds, featuring ring-expansion reactions, including [2 + 2] cycloaddition/cyclobutene fragmentation and acylation/ring-expansion sequences.

As previously noted, small-molecule libraries with medium-sized rings are quite rare because of the challenges of synthesizing complex structures similar to natural products. To address these limitations, Tang et al. introduced a novel site-selective functionalization and ring-expansion strategy for converting small-ring structures in polycyclic frameworks into medium-sized rings in 2019 [83]. This strategy aimed to diversify natural products and generate a collection of compounds, navigating the largely untapped chemical space of polycyclic compounds with medium-sized rings. The approach is distinguished by a two-phase process: steroid diversification, which involves the site-selective oxidation of C–H bonds in polycyclic natural products to form C–O bonds, followed by ring expansion using these C–O bonds or native C–O bonds (Scheme 8a). C–H functionalization reactions, particularly C–H oxidation, have emerged as a vital method for modifying complex organic molecules [84,85,86,87]. Among these, electrochemical oxidation is particularly noteworthy for producing minimal toxic waste and for its broad compatibility with various functional groups [88]. This makes it a superior choice over traditional chemical methods. By adopting Baran’s electrochemical allylic C–H oxidation method [89,90] coupled with a Beckmann rearrangement, this approach facilitates the synthesis of a range of lactams with expanded rings **81**, demonstrating its effectiveness in enhancing the structural diversity of organic molecules (Scheme 8b). Moreover, using site-selective Cu-mediated C–H oxidation [91] combined with a Beckmann rearrangement, the C-ring in estrone was successfully expanded (Scheme 8c). Further expanding the scope for complex structural transformations using C–O bonds, this method incorporates a variety of ring-expansion methods, including [2 + 2] cycloaddition and subsequent cyclobutene fragmentation, as well as an acylation/ring-expansion sequence developed by Unsworth et al. (Scheme 8d) [73,74,75,76,77,78,79]. The creation of 150 compounds, characterized by their intricate, natural-product-like structures with polycyclic ring systems, sp^3^ hybridized carbons, and a wealth of stereogenic centers, highlights the effectiveness of this approach. The diversity of these compounds emphasizes their potential for use in varied biological processes, attributed to their uniquely structured medium-sized rings.

## 6. Conclusions

Natural products, with their diverse and intricate structures, are prime candidates for bioactivity screening; however, their syntheses are often met with challenges. These challenges prevent us from fully exploiting the therapeutic potential of such compounds, driving the need for innovative synthetic approaches. Despite significant strides in DOS, Ctd, BIOS, pseudo-natural product synthesis, pDOS, and biomimetic methods, the creation of small-molecule libraries that accurately mimic the complexity of natural products, particularly those with medium-sized rings, remains difficult. This review has highlighted novel ring-expansion strategies as a response to these synthetic challenges, emphasizing their importance in the efficient creation of medium-sized rings. These strategies are crucial not only for enhancing the structural complexity and diversity of small-molecule libraries but also for facilitating new breakthroughs in drug development. The potential of ring-expansion reactions to generate novel bioactive compounds is immense. Future research could explore uncharted chemical spaces, harnessing the power of these reactions to discover unique structures with unknown pharmacological activities. Additionally, integrating ring-expansion reactions with other emerging synthetic methods could offer unprecedented routes to complex molecules, leading to the identification of bioactive compounds for undruggable targets.

## Data Availability

Not applicable.
